# Regulatory CD4^+^Foxp3^+^ T Cells Control the Severity of Anaphylaxis

**DOI:** 10.1371/journal.pone.0069183

**Published:** 2013-07-26

**Authors:** Reem Kanjarawi, Michel Dy, Emilie Bardel, Tim Sparwasser, Bertrand Dubois, Salah Mecheri, Dominique Kaiserlian

**Affiliations:** 1 CIRI, International Center for Infectiology Research, “Mucosal immunity, Vaccination & Biotherapies” Team, Université de Lyon, Lyon, France; 2 INSERM, U1111, Lyon, France; 3 École Normale Supérieure de Lyon, Lyon, France; 4 Université Lyon 1, Centre International de Recherche en Infectiologie, Lyon, France; 5 CNRS, UMR5308, Lyon, France; 6 CNRS/UMR 8147, université René Descartes, Hôpital Necker, Paris, France; 7 Institute of Infection Immunology, TWINCORE, Center for Experimental and Clinical Infection Research, Hannover, Germany; 8 Institut Pasteur, Unité de Biologie des Interactions Hôte Parasites, Paris, France; 9 Centre National de la Recherche Scientifique, Unité de Recherche Associée 2581, Paris, France; University of Nebraska Medical center, United States of America

## Abstract

**Objective:**

Anaphylaxis is a life-threatening outcome of immediate-type hypersensitivity to allergen, consecutive to mast cell degranulation by allergen-specific IgE. Regulatory T cells (Treg) can control allergic sensitization and mast cell degranulation, yet their clinical benefit on anaphylactic symptoms is poorly documented. Here we investigated whether Treg action during the effector arm of the allergic response alleviates anaphylaxis.

**Methods:**

We used a validated model of IgE-mediated passive systemic anaphylaxis, induced by intravenous challenge with DNP-HSA in mice passively sensitized with DNP-specific IgE. Anaphylaxis was monitored by the drop in body temperature as well as plasma histamine and serum mMCP1 levels. The role of Treg was analyzed using MHC class II-deficient (Aβ^°/°^) mice, treatment with anti-CD25 or anti-CD4 mAbs and conditional ablation of Foxp3^+^ Treg in DEREG mice. Therapeutic efficacy of Treg was also evaluated by transfer experiments using FoxP3-eGFP knock-in mice.

**Results:**

Anaphylaxis did not occur in mast cell-deficient W/W^v^ mutant mice and was only moderate and transient in mice deficient for histamine receptor-1. Defects in constitutive Treg, either genetic or induced by antibody or toxin treatment resulted in a more severe and/or sustained hypothermia, associated with a rise in serum mMCP1, but not histamine. Adoptive transfer of Foxp3^+^ Treg from either naïve or DNP-sensitized donors similarly alleviated body temperature loss in Treg-deficient DEREG mice.

**Conclusion:**

Constitutive Foxp3^+^ Treg can control the symptomatic phase of mast cell and IgE-dependent anaphylaxis in mice. This might open up new therapeutic avenues using constitutive rather than Ag-specific Treg for inducing tolerance in allergic patients.

## Introduction

Anaphylaxis is a severe life-threatening systemic immediate-type hypersensitivity reaction to dietary or respiratory allergens, characterized by vasodilation, bronchoconstriction, hypotension, hypothermia, and sometimes death within minutes or hours after re-exposure to the allergen in sensitized individuals [1]. Allergic sensitization (i.e. the asymptomatic phase) results from the priming of Th2 cells and the production of specific IgE, which bind to the high affinity FcεRI receptor expressed by tissue mast cells (MC). In susceptible patients, re-exposure to the allergen causes the cross-linking of specific IgE and aggregation of cell surface FcεRI complexes. This leads to the symptomatic effector phase of anaphylaxis, induced MC degranulation and immediate release of preformed mediators such as histamine, mouse mast cell protease-1 (mMCP-1) [2], trypase [3] and platelet-activating factor (PAF) [4] and *de novo* synthesis of inflammatory mediators such as leukotrienes, prostaglandins and cytokines [5].

Regulatory T cells (Treg) including constitutive and Ag-induced Foxp3^+^ Treg appear to be essential for controlling adaptive immunity and expression of autoimmune and allergic diseases [6,7]. In humans, their crucial role is exemplified in patients with X-linked immune dysregulation polyendocrinopathy (IPEX) syndrome, in whom mutations in the transcription factor FOXP3, cause a fatal autoimmune and allergic phenotype [8], and polymorphisms are associated with atopy development during childhood [9]. Defects in the number and function of CD4^+^CD25^+^ Treg were also reported in patients suffering from asthma [10] or rhinitis [11]. In addition, selective depletion of Foxp3^+^ Treg before allergen sensitization exacerbates specific IgE, Th2-type cytokine responses and eosinophil lung infiltration [12], emphasizing a role for constitutive Foxp3^+^ Treg in the priming phase of the allergic response. Besides, allergen-induced adaptive Treg could account for tolerance induction in already sensitized individuals [13]. Recently, the protective effect of a non-depleting anti-CD4 mAb, when administered during sensitization but not challenge, was proposed to be mediated by Treg [14]. However, there is as yet little evidence that Treg action during the symptomatic phase of anaphylaxis, can improve severity or duration of symptoms.

Several mechanisms may contribute to Treg-mediated suppression of allergic diseases: i) reduced priming of allergen-specific Th2-type T cells [11,15,16] and B cells [17], ii) switch from allergen-specific IgE to IgG_4_ production [18] and iii) downregulation of MC degranulation via inhibition of calcium influ*x* [19] or expression of FcεRI [20]. Thus, evidence that Foxp3^+^ Treg can control the symptomatic effector phase of immediate-type hypersensitivity and attenuate anaphylaxis remains scarce [21]. In this respect, we recently documented that anti-CD25 mAb injection before oral sensitization with a food allergen, enhanced both the priming of allergen-specific T and B cells and the severity of immediate-hypersensitivity symptoms after oral challenge [22]. Importantly, when the anti-CD25 mAb was injected just before challenge, we observed enhanced serum levels of mMCP1 [22] and more numerous degranulated intestinal MC (D. Kaiserlian, *unpublished data*), suggesting a role for Treg on tissue MC cell degranulation during the symptomatic phase of anaphylaxis.

To specifically address the role of Foxp3^+^ Treg in clinical manifestation of allergy, we used a model of passive systemic anaphylaxis (PSA), which reproduces the symptomatic phase of immediate-type hypersensitivity. We show here in four settings of Treg deficiency that constitutive Foxp3^+^ Treg can control the severity and duration of the anaphylactic shock, as determined both by hypothermia and serum mMCP1 level.

## Materials and Methods

### Mice

Female C57BL/6 (B6) mice (6 weeks old) were purchased from Charles River Laboratories (L’Arbesle, France). MHC class II-deficient (Aβ^°/°^) mice were kindly provided by D. Mathis and C. Benoist. Mast cell-deficient WBB6F1-homozygous mutants (W/W^v^ ,Ho), heterozygous (W/W^v^, He) and wild type littermates (+/+) were kindly provided by Dr. Salah Mécheri (Pasteur Institute, Paris, France). H1R^°/°^ mice were kindly provided by T. Watanabe [23]. Foxp3-eGFP knock-in (KI) mice on the B6 background [24] were kindly provided by B. Malissen (CIML, Marseille, France). DEREG mice (DEpletion of REGulatory T cells) expressing both a diphtheria toxin receptor (DTR) and the eGFP fusion protein under the control of the *foxp3* locus have been described [25]. These mice allowed for both detection of eGFP ^+^ Foxp3^+^ Treg by flow cytometry and depletion of Foxp3^+^ Treg by DT injection. All mice were housed in our animal facility in specific pathogen-free (SPF) conditions, and used between six to ten weeks of age.

All animal work and experimental procedures have been conducted according to relevant national and international guidelines, with special attention to minimize animal pain and have been approved by our local Institutional Animal Care and Use Committee, CECCAP («Comité d’Evaluation Commun au Centre Léon Bérard, à l’Animalerie de transit de I’ENS, au PBES et au laboratoire P4») (protocol reference number: ENS_2011_022).

### Passive Systemic Anaphylaxis (PSA)

Systemic anaphylaxis was induced in mice by *i.v* injection of anti-DNP IgE (10 µg) (clone SPE-7) (SIGMA-Aldrich, L’Isle d’Abeau, France). Twenty-four hours later, mice were challenged by *i.v* injection of 2, 4, dinitro-phenyl conjugated to human serum albumin (DNP-HSA) (SIGMA-Aldrich, L’Isle d’Abeau, France) (500 µg) or PBS as control. Systemic anaphylaxis was evaluated by hypothermia, measured by body temperature every ten minutes during 1 h after challenge, using a rectal probe coupled to a digital thermometer (Physitemp Instruments INC, Phymep, Paris, France).

### Histamine titration

Plasma was collected from mice 1,5 minute after challenge with DNP-HSA. Histamine levels were quantified using HTRF-based immunoassay (Homogeneous Time-Resolved Fluorescense), according to the manufacturer’s instruction (Cisbio bioassays, Bagnols-sur-Cèze, France). Briefly, samples or histamine calibrator were incubated with acylated buffer and reagent during 15 minutes at room temperature. Allophycocyanine (XL665)-labelled histamine and anti-histamine conjugated to Cryptate were then added and incubated for 3 h at room temperature. The fluorescence ratio (665nm/620nm) was read using HTRF compatible reader. The specific signals are inversely proportional to the concentration of acylated histamine in the samples.

### Mouse mast cell protease titration

mMCP-1 was titrated in serum collected at 1hr after challenge by ELISA using the Moredun Kit (Moredun Scientific Ltd, Midlothian, UK) according to the manufacturer instructions. Briefly, Maxisorp plates (Nunc, Roskild, Denmark) coated with 2µg/ml of Sheep anti-mMCP-1 capture Ab were incubated with serial 10 fold dilutions of serum. Specific binding was detected using peroxidase-labelled Rabbit anti-mMCP-1 Ab and Tetramethylbenzidine (KPL, Gaithersburg, USA) as substrate. OD was read at 450 nm and levels of mMCP-1 (expressed in ng/ml) were calculated from the standard curve obtained using recombinant mMCP1 diluted in normal mouse serum.

### Depletion of regulatory T cells

C57BL/6 mice were injected intraperitonealy (*i.p*) with either 500 µg of the anti-CD25 mAb (clone PC61.5.3-Rat IgG1, BioXcell, USA) or anti-CD4 mAb (clone GK 1.5-Rat IgG2b, BioXcell) one day before sensitization with anti-DNP IgE. For conditional ablation of Foxp3^+^ Treg, DEREG mice were injected i.p with 1 µg of diphtheria toxin (DT) one day before IgE sensitization [25]. Efficacy of depletion of Foxp3^+^ Treg was > 99%, as shown by FACS analysis (Figure S1): on day 2 after DT injection, peripheral lymph nodes (axillary and inguinal) were stained with PE-conjugated anti-CD25 mAb (clone PC61), PerCP-Cy5.5-conjugated anti-CD4 mAb (clone RM4-5) both from (BD Pharmingen, San Diego, California) and Foxp3 expression was analyzed by GFP expression.

### Transfer of Foxp3^+^ Treg in DEREG mice

Peripheral lymph nodes (axillary, inguinal) were isolated from Foxp3-eGFP KI mice either naïve or at day 6 after skin painting with 0.5% of 2, 4, dinitro-fluorobenzene (DNFB) diluted in acetone/olive oil (4: 1 v/v). CD4^+^ T cells were first enriched by positive selection using anti-CD4 mAb -coated microbeads (Miltenyi Biotec), then Foxp3^+^-eGFP^+^ Treg were isolated by FACS sorting, with purity routinely > 92%. Two million of CD4^+^Foxp3^+^ cells from either naïve or DNFB-sensitized Foxp3-eGFP KI mice were transferred *i.v* to DEREG mice one day after DT injection. One day after transfer, all mice were passively sensitized with DNP-specific IgE (10 µg) and challenged with DNP-HSA (500 µg).

### Statistical analysis

Statistical analyses were performed using the two-way ANNOVA test for kinetics of body temperatures and the Man-Whitney nonparametric test for histamine and mMCP1 levels. p values < 0.05 were considered statistically significant.

## Results

### 1: Passive systemic anaphylaxis (PSA) depends on mast cells and histamine signaling

**Figure 1:**
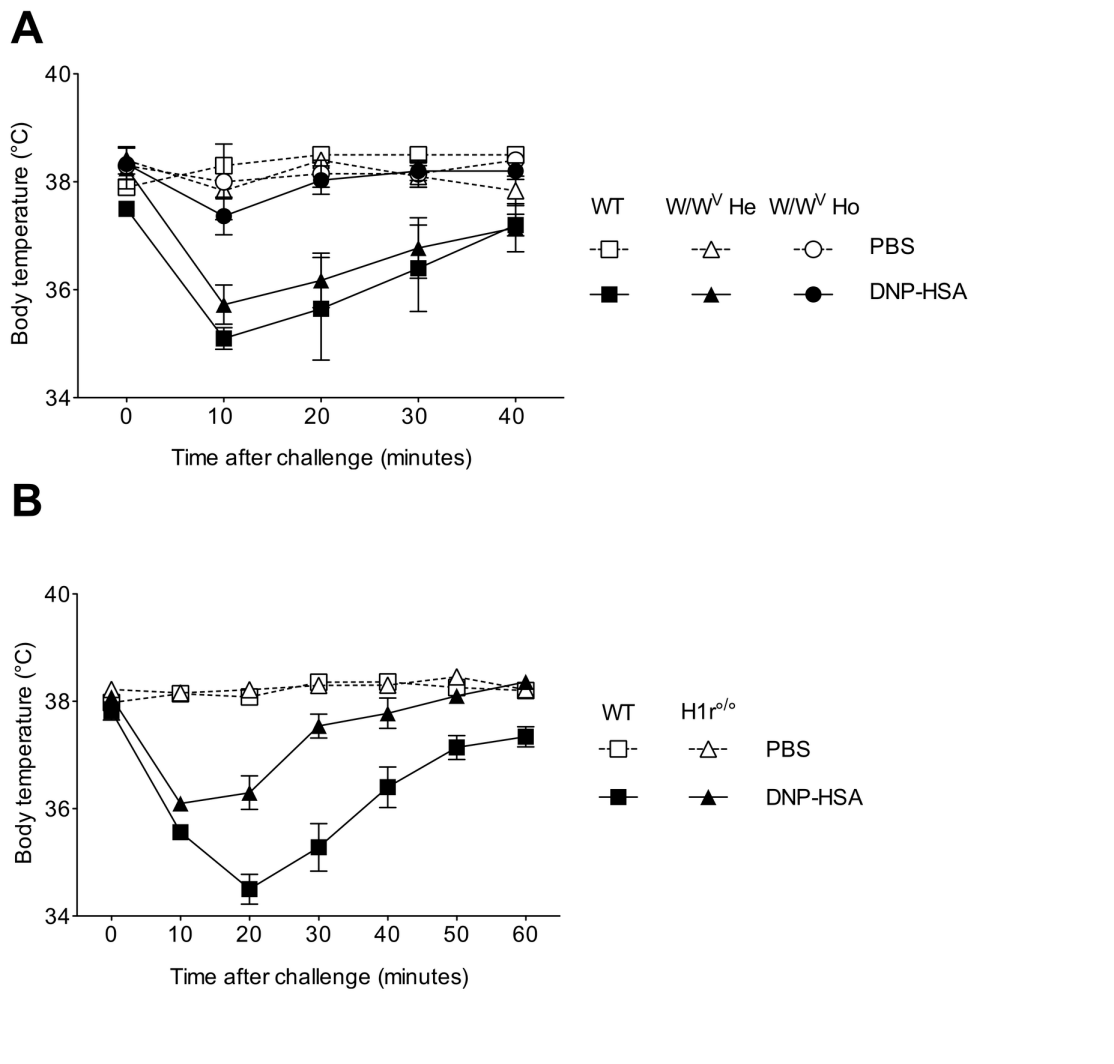
Passive systemic anaphylaxis (PSA) depends on mast cells and histamine. PSA was induced by *i.v.* sensitization with 10 µg of anti-DNP IgE and challenge 24hrs later with 500 µg of DNP-HSA (*black symbols*) or PBS as control (*white symbols*) in MC-deficient mice homozygotes mutants (W/W^v^ Ho) (*circles*), heterozygotes (W/W^v^ He) (*triangles*) and their WT littermates (+/+) (*squares*) (A) and in Histamine receptor-1-deficient (HR1^°/°^) (*triangles*) and WT B6 mice (*squares*) mice (B). Body temperature was measured at various time points after challenge. Data are presented as mean (± SEM) of body temperature (n= 5 mice/group).

We first verified whether MC were implicated in the induction of DNP-specific IgE-mediated PSA. As expected, DNP-HSA challenge of IgE-sensitized MC-sufficient mice (W/W^v^ heterozygotes (W/W^v^ He) and wild type littermates) caused a rapid and intense decline in body temperature that was maximal at 10 min post challenge and progressively recovered to a normal temperature at 40 min. In contrast, the body temperature of MC-deficient homozygote (W/W^v^ Ho) mutant mice remains unaffected throughout the time course of the protocol (P = 0.0292) (Figure 1A). As expected a rise in plasma histamine levels at 1,5 min post challenge was observed in MC-sufficient, but not MC-deficient mice (*data not shown*). Since histamine is a preformed MC mediator, chiefly involved in the immediate symptoms of anaphylaxis mainly via binding to histamine H1 receptors [26], we investigated the severity of anaphylaxis in H1R^°/°^ mice. We found that the drop in body temperature was only moderate and transient in H1R^°/°^ as compared to WT B6 control mice (p=0.0027) (Figure 1B). Altogether, these data confirm that anaphylaxis, as measured by hypothermia, depends on MC and histamine.

### 2: Defect in MHC class II-restricted CD4^+^ CD25^+^ T cells aggravates anaphylaxis

**Figure 2:**
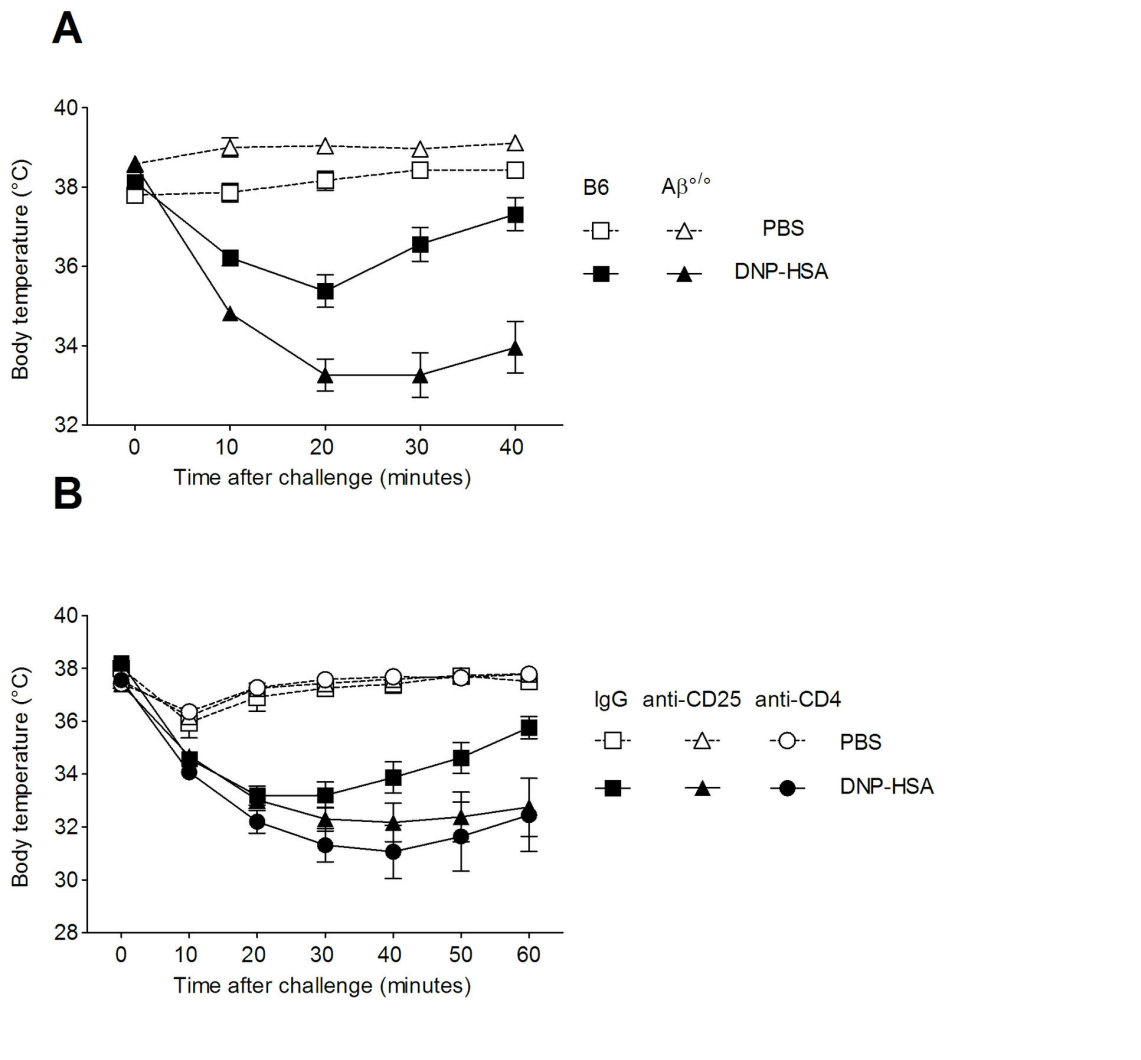
Analysis of anaphylaxis in settings of CD4^+^CD25^+^ T cell deficiency. DNP-specific PSA was induced A) in MHC class II-deficient mice (Aβ^°/°^) (*triangles*) and WT B6 (*squares*) mice and B) in B6 mice injected with either an anti-CD25 mAb (*triangles*), an anti-CD4 mAb (*circles*) or a control rat IgG mAb (*squares*). All mice were passively sensitized with anti-DNP IgE and challenge with DNP-HSA (*black symbols*) *or* PBS (*open symbols*). Data show mean (± SEM) values of body temperature at various times after challenge. Figure 2A shows 1 representative experiment out of 3 (n= 5-6 mice/group); p** = 0,0027 for DNP-challenged Aβ KO versus WT B6 (p** = 0,0074 and p = * 0,0286, for the other two experiments). Figure 2B data are from 2 pooled experiments (n= 5 mice/group); p* = 0,017 for DNP-challenged WT B6 versus anti-CD4-mAb treated mice and p* = 0,05 for DNP-challenged WT versus anti-CD25 mAb-treated B6 mice.

To study the role of Treg in anaphylaxis, we investigated the severity of IgE-mediated PSA in 3 different settings of Treg deficiency: MHC class II-deficient (Aβ^°/°^) mice lacking MHC class II-restricted CD4^+^ T cells [27], WT B6 mice treated with anti-CD25 or anti-CD4 depleting mAbs and ablation of Foxp3^+^ Treg by DT injection in Foxp3-DTR transgenic DEREG mice. We first observed that, as compared to syngeneic WT B6 mice, Aβ^°/°^ mice exhibited upon DNP-HSA challenge a more severe decline in body temperature that was detected as early as 10 min post challenge and was sustained up to 40 min after challenge (p ** = 0.0027) (Figure 2A). This indicated that MHC class II^+^ cells and/or class-II-restricted CD4^+^ T cells are involved in the negative regulation of anaphylaxis.

To investigate whether CD4^+^CD25^+^ Treg contributed to alleviation of anaphylaxis, PSA was tested in B6 mice that were injected with the depleting anti-CD25 mAb or the anti-CD4 mAb, 24h before anti-DNP IgE sensitization. FACS analysis in lymphoid organs showed that anti-CD25 mAb treatment resulted in >90% reduction of CD4^+^CD25^+^ Treg frequency with concomitant increase in the percentage of Foxp3^+^ CD25^-^ T cells (*not shown*), indicative of a down-regulation of the CD25 molecule previously described to render Treg functionally inactive [22,28]. Anti-CD4 mAb-treated mice showed nearly complete (>99%) depletion of CD4^+^ T cells (*not shown*). Analysis of body temperature after DNP-HSA challenge showed that, as compared to control IgG-injected mice, anti-CD25 mAb-treated mice exhibited sustained hypothermia up to 60 min after challenge (p=0,05), while anti-CD4 mAb-treated mice showed both a more severe and sustained loss of body temperature (p=0,017) (Figure 2B). These data indicate that CD4^+^ CD25^+^ T cells negatively control the severity of anaphylaxis.

### 3: Ablation of Foxp3^+^ Treg in DEREG mice induced severe and sustained anaphylaxis

**Figure 3:**
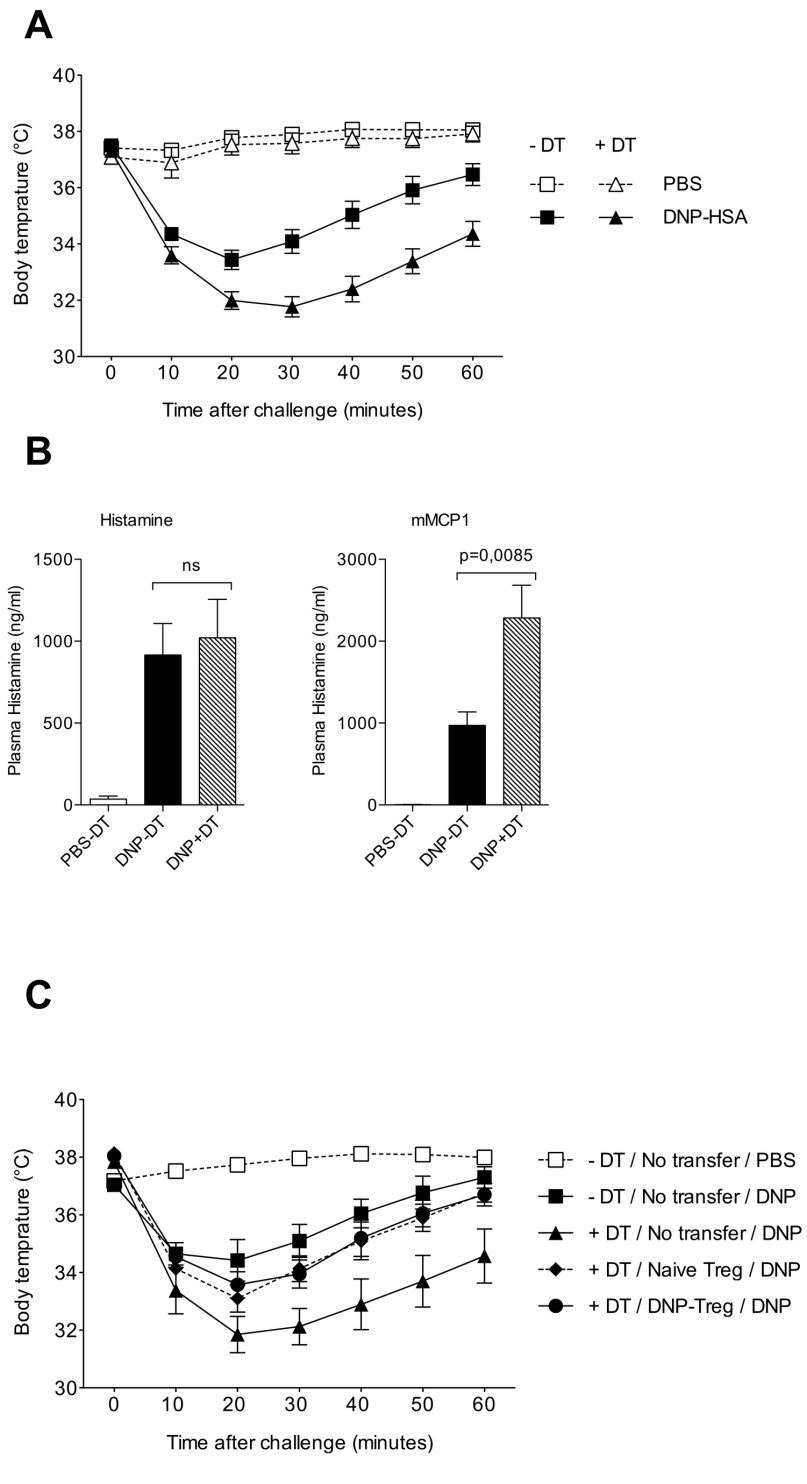
Foxp3^+^ Treg control the severity and duration of anaphylaxis symptoms. **A**: DEREG mice were injected with 1µg of DT (*triangles*) or with PBS (*squares*) on day -1, injected with anti-DNP IgE on day 0 and challenged with DNP-HSA (*black symbols*) or PBS (*white symbols*) on day +1. Body temperature was measured at various times after challenge. The data show pooled values from 3 experiments using 4-6 mice/group in each. p** = 0,0007 (2 way ANOVA) for DNP-challenged DEREG with and without DT (p values for each independent experiments are p= 0,032; p= 0,0176 and p=0,0105). **B**: plasma histamine (*left panel*) and serum mMCP-1 (*right panel*) were titrated in IgE-sensitized DEREG mice that were DT-untreated (black bars) or DT-treated (*grey bars*). IgE-sensitized and PBS-challenged DT-untreated DEREG mice (*white bars*) were used as negative controls. Data show mean ± SEM values (n= 8-10 mice/group) of each factor expressed as ng/ml. Statistical analysis by 2 way ANOVA comparing DEREG with and without DT show significance with p** = 0,0085. **C**: DT-untreated (*white symbol*) or DT-treated (*black symbols*) DEREG mice were either un-transferred (*black triangles*) or transferred with CD4^+^Foxp3^+^ Treg from either naïve mice (*black diamond*) or from day 6 DNFB-sensitized mice (*black circles*). Mice were passively sensitized with IgE anti-DNP, challenged with DNP-HSA as indicated and body temperature was measured at various times after challenge. Data show mean (± SEM) of body temperature using 5 mice/group.

To determine whether complete ablation of Foxp3^+^ Treg aggravates symptoms of anaphylaxis, we used DEREG mice that express the DT receptor under the control of the *Foxp3* promoter [25]. As expected, DT injection depleted more than 98% of CD4^+^ Foxp3^+^ Treg including both CD25^+^ and CD25^-^ cells, as determined by FACS analysis of inguinal and axillary LN cells (Figure S1). In control PBS-treated DEREG mice, IgE sensitization and allergen challenge caused a significant drop in body temperature that was maximal at 20 min post challenge, with progressive recovery by 60 min post challenge (Figure 3A). Anaphylaxis was associated with a brisk rise in plasma histamine levels at 1.5 min after challenge as well as with mMCP-1 release at 1h after challenge (Figure 3B). Importantly, DT-treated DEREG mice exhibited a more pronounced and persistent decrease in body temperature, as compared to DT-untreated DEREG mice (p** = 0.0007) (Figure 3A) upon PSA induction. Although enhanced anaphylaxis in Treg depleted DEREG mice was not accompanied by histamine increase (Figure 3B, left panel), it did associate with enhanced mMCP-1 levels in serum (Figure 3B, right panel, p** = 0,0085). These data emphasize that constitutive Foxp3^+^ Treg control the intensity and duration of hypothermia during anaphylactic shock, and that this is correlated with inhibition of MC degranulation as assessed by measuring serum mMCP-1 levels.

### 4: Adoptive transfer of Foxp3^+^ Treg reduce the severity of anaphylaxis in DEREG mice

We then analyzed whether the severe anaphylaxis in Foxp3^+^ Treg-deficient DEREG mice could be alleviated by adoptive transfer of Foxp3+ Treg. To this end we compared the therapeutic benefit of constitutive Foxp3^+^ Treg isolated from naïve mice to that of Foxp3^+^ Treg isolated from DNP-immunized mice, which contain both constitutive Foxp3^+^ Treg and a subset of highly suppressive allergen-specific ICOS ^+^ Foxp3^+^ Treg [29]. CD4+eGFP+ T cells were FACS-sorted from lymph nodes of either naïve or day 6 DNFB-skin sensitized Foxp3-eGFP KI mice and transferred to DT-treated DEREG mice one day before IgE sensitization. As shown before, DT injection in DEREG mice aggravated anaphylaxis after DNP-HSA challenge. Importantly, DT-treated DEREG mice no longer exhibited such enhanced hypothermia, when reconstituted with either constitutive or DNP-induced CD4^+^Foxp3^+^ Treg, resulting in both cases in severity and duration of hypothermia that was comparable to that of Treg sufficient (non DT-injected) DEREG mice Figure 3C). These data show that Foxp3^+^ Treg from naïve and DNP-sensitized donors exert similar beneficial effect on body temperature drop, indicating that constitutive Foxp3^+^ Treg may be sufficient to alleviate anaphylaxis.

## Discussion

Substantial evidence from experimental animal models have documented the capacity of Treg to control allergic diseases by down-regulating the priming of allergen-specific Th2- or Th1-type responses during the asymptomatic sensitization phase. Yet, whether Treg can inhibit the symptomatic effector arm of the allergic response remains poorly documented. A major bottleneck to specifically address this issue is the fact that anti-CD25 mAb depletion strategies do not allow to study the role of Treg at various phases of the allergic responses, since CD25 is also expressed on non Treg cells (e.g. B cells, activated T cells and dendritic cells), which in already sensitized individuals may all contribute to the allergic response induced by allergen provocation.

In the present study we took advantage of a well-validated model of PSA, to selectively study the sole effector phase of allergy and exploited DEREG mice to address the role of constitutive Foxp3^+^ Treg and evaluate by transfer experiments the relative beneficial effect of constitutive versus allergen-specific Foxp3^+^ Treg on MC-mediated allergic symptoms. We document that MHC class-II restricted CD4^+^CD25^+^Foxp3^+^ Treg play a protective role in IgE-mediated systemic anaphylaxis in mice. This was demonstrated in different experimental settings including i) genetically deficient Aβ^°/°^ mice, deficient in MHC class II-restricted CD4^+^ T cells ii) antibody depletion of CD4^+^ T cells or inactivation of CD25^+^ Treg and iii) selective ablation of Foxp3^+^ Treg by DT injection in DEREG mice. In all these settings, hypothermia, which is the gold standard clinical sign of severe anaphylaxis in mice [30,31], was exacerbated. It should be noted however that hypothermia was more sustained in anti-CD25 mAb treated mice and Aβ^°/°^ mice compared to DT-injected DEREG mice. The reason for faster recovery after DT injection is unclear at present but could be related to *in vivo* persistence of the injected anti-CD25 mAb causing long lasting depletion as compared to a more transient effect of DT. Alternatively, it could reflect a bystander effect of DT on cells/pathways positively contributing to anaphylaxis. Moreover, we showed that adoptive transfer of Foxp3^+^ Treg, from either naïve or DNP-sensitized mice, into DT-treated DEREG mice similarly decreased the severity of this typical clinical symptom and accelerated recovery, supporting the therapeutic value of constitutive Foxp3^+^ Treg.

In this study, we provided evidence that Foxp3^+^ Treg can exert a physiological control of hypothermia, a major systemic symptom of anaphylaxis. Because the PSA mouse model solely probes the efferent phase of immediate type hypersensitivity, which critically requires MC [4] (*and our present data*), it is likely that Treg alleviate anaphylaxis by suppressing MC effector function. This conclusion is indeed supported by our observation that the exacerbated hypothermia in Treg-depleted DEREG mice is associated with increased serum levels of mMCP1, an immediate mediator of tissue MC, which is produced in MC-sufficient but not MC-deficient W^v/^ W^v^ Ho mice (*data not shown*). Surprisingly, although we found a brisk rise of plasma histamine at 1,5 min post challenge, histaminemia was not increased in the absence of Treg, irrespective of the model of Treg deficiency we used (*data not shown* and Figure 3B left panel). That anaphylaxis-associated hypothermia can be modulated without any change of histamine levels is reminiscent to another study using the same model of IgE-mediated PSA, in which reduced hypothermia induced by IL-10 overexpression was not matched by changes in serum histamine [32]. Previous studies, using anti-CD25 mAb infusion and CD4^+^CD25^+^ T cell transfer in a similar IgE-dependent PSA model in B6 mice, documented that CD4^+^CD25^+^ T cells reduced plasma histamine level *in vivo* [19]. Unfortunately, whether such modulation of histamine levels by Treg has any clinical outcome on hypothermia or other symptoms of anaphylaxis was not reported. Yet, such discrepancy in the effect of Treg on histamine level could be due to subtle differences in the experimental procedures including the dose of IgE used for sensitization, the transient nature of plasma histamine release and environmental factors pertaining to animal housing conditions such as the microbial environment and stress conditions. It is therefore conceivable that Treg modulate PSA by inhibiting MC production of mediators other than histamine. Whether Treg modulate other mediators besides mMCP1, such as PAF, which contribute also to the classical IgE pathway of anaphylaxis [4], will require further investigations. In any case, our data illustrate that the follow-up of body temperature is a more reliable readout compared to changes in plasma histamine levels to detect the physiological impact of Treg on allergic symptoms.

Several direct or indirect mechanisms could account for the regulatory effect of Treg on MC during the efferent phase of IgE dependent anaphylaxis. Treg could interact with MC to control their effector function. Indeed, MC and Treg were described to co-localize in lymphoid organs [19] and *in vitro* culture experiments revealed that Treg can down-regulate FcεRI-induced release of β-hexosaminidase by MC, by a cell contact-dependent mechanism involving OX40/OX40-L interactions and leading to a cAMP-dependent reduction of Calcium influx [19]. Other studies showed that Treg decreased MC FcεRI expression and could also interfere with the MC signaling cascade downstream internalization of FcεRI/Allergen complexes [20]. In this context, IL10 might be a good candidate molecule in Treg-mediated control of anaphylaxis. Indeed, this cytokine can be produced by foxp3^+^ Treg [33] and its overexpression in mice leads to decreased FcεRI expression on MC and attenuated hypothermia during PSA [14]. A definitive answer to this issue requires further investigation. Alternatively, Treg may indirectly affect MC function involved in the triggering of PSA immediate symptoms. Indeed, Foxp3 Treg have the capacity to interact with the skin vasculature and to alleviate allergic contact dermatitis by regulating the extravasation of CD8 effector T cells into the skin [34]. In addition, an elegant study recently revealed that skin MC (at variance to peritoneal MC), express unsaturated FcεRI, are distributed perivascularly and can actively sample intravascular IgE by extending cell processes across vessels, by a mechanism independent of histamine secretion [35]. It is thus conceivable that Treg in the vicinity of blood vessels irrigating the skin or mucosal tissues, could somehow limit the active uptake of circulating IgE by perivascular MC, thereby resulting in decreased uploading of the systemically injected allergen DNP-HSA and reduced severity signs of immediate-type allergy.

Interestingly, transfer of constitutive Foxp3^+^ Treg to DEREG mice appeared sufficient to improve systemic symptoms of allergy and accelerate clinical recovery, suggesting that their suppressive efficacy is either T cell-receptor independent or involves T cell receptor activation by self or endogenous ligands. This is supported by the observation that similar clinical improvement was achieved by transfer of either constitutive Foxp3^+^ Treg from naïve mice or Foxp3^+^ Treg originating from DNP-sensitized mice, although they also contain highly suppressive Ag-specific ICOS^+^ Treg [29]. These findings extend a previous study in a model a food allergy showing that CD25^+^ T cell transfer before allergen challenge can alleviate the clinical signs of anaphylaxis [36] and indicate that constitutive Foxp3^+^ Treg are sufficient to control the efferent phase of allergic reaction mediated in large part by MC.

Altogether, these data suggest that differences in the number, fate or function of constitutive Foxp3^+^ Treg may reflect the differential susceptibility to allergen provocation amongst allergic individuals and support the idea that strategies re-inforcing the survival, number or function of constitutive Foxp3^+^ Treg may be a valuable approach for immunotherapy of immediate-type allergic reactions.

## Supporting Information

Figure S1 Efficacy of Treg depletion by DT injection in DEREG mice. DEREG mice were injected with DT (or PBS as control) one day before IgE anti-DNP mAb injection, and the frequency of Foxp3^+^ Treg was assessed 2 days later by FACS analysis using anti-CD4-PercP-Cy5.5 and anti-CD25-PE conjugates and Foxp3 was determined by GFP expression. The dot plots show the percentage of CD25^+^/Foxp3^+^ cells in gated CD4^+^ T cells.
(TIF)
